# A novel mechanism of iron-core formation by *Pyrococcus furiosus* archaeoferritin, a member of an uncharacterized branch of the ferritin-like superfamily

**DOI:** 10.1007/s00775-012-0913-0

**Published:** 2012-06-28

**Authors:** Kourosh Honarmand Ebrahimi, Peter-Leon Hagedoorn, Laura van der Weel, Peter D. E. M. Verhaert, Wilfred R. Hagen

**Affiliations:** Department of Biotechnology, Delft University of Technology, Julianalaan 67, 2628BC Delft, The Netherlands

**Keywords:** Ferritin-like superfamily, Ferroxidase, Iron, Storage, Mass spectrometry

## Abstract

Storage of iron in a nontoxic and bioavailable form is essential for many forms of life. Three subfamilies of the ferritin-like superfamily, namely, ferritin, bacterioferritin, and Dps (DNA-binding proteins from starved cells), are able to store iron. Although the function of these iron-storage proteins is constitutive to many organisms to sustain life, the genome of some organisms appears not to encode any of these proteins. In an attempt to identify new iron-storage systems, we have found and characterized a new member of the ferritin-like superfamily of proteins, which unlike the multimeric storage system of ferritin, bacterioferritin, and Dps is monomeric in the absence of iron. Monomers catalyze oxidation of Fe(II) and they store the Fe(III) product as they assemble to form structures comparable to those of 24-meric ferritin. We propose that this mechanism is an alternative method of iron storage by the ferritin-like superfamily of proteins in organisms that lack the regular preassociated 24-meric/12-meric ferritins.

**Electronic supplementary material:**

The online version of this article (doi:10.1007/s00775-012-0913-0) contains supplementary material, which is available to authorized users.

## Introduction

Iron is involved in many biocatalytic reactions and electron transport processes in the cell [1]. At physiological pH and in the presence of molecular oxygen, the reactivity of free Fe(II) can cause fundamental problems. The reaction with O_2_ results in two products: (1) reactive oxygen species (ROS) such as hydroxyl and superoxide radicals that can damage components of cells and (2) Fe(III), which is essentially insoluble at physiological pH, with a solubility of 10^−10^ M [2]. The molecular machinery of living organisms is required to store, transport, and deliver iron in a safe manner. Storage of iron in a nontoxic form as iron(III) oxide mineral nanoparticles has been reported for ferritin, bacterioferritin, and Dps (DNA-binding proteins from starved cells), which are members of the ferritin-like superfamily of proteins. This superfamily consists of at least six other known subfamilies, including rubrerythrin, manganese catalase, methane monooxygenase, ribonucleotide reductase small chain, fatty acid desaturase 2, and the transfer RNA modifying enzyme MiaE [3]. All known members of this family are characterized by the presence of a four α-helix bundle domain [3] with a dimetal binding site in the middle, with the exception of Dps, in which the dimetal binding site is located at the interface between two subunits.

Ferritin has been found in bacteria [4], archaea [5], plants [6], stramenopiles [7], and vertebrates [8]. 24 subunits assemble to make a hollow spherical protein with octahedral symmetry (432 symmetry) and a molecular mass of 480 kDa. Ferritin binds and oxidizes Fe(II) via a process that is still not fully understood, and subsequently stores the Fe(III) product inside its cavity as a ferrihydrite-like mineral core. In vitro it can store up to about 3,000 iron atoms per protein molecule [9, 10]. Mammalian ferritin consists of two different subunits: H (“heavy” chain) and L (“light” chain) [11]. A third subunit, M (“medium” chain), has been found in bullfrog ferritin [12]. In contrast, bacterial and archaeal ferritins consist of one subunit type [5, 13]. A second subfamily of the ferritin-like superfamily that is able to store iron is bacterioferritin, which has only been found in bacteria [14]. It is very similar to ferritin, but unlike ferritin it has a heme group at the interface of each of the two subunits. Fast oxidation and mineralization of Fe(II) by 24-meric ferritins and bacterioferritins requires a diiron binding site, the ferroxidase center (FC) [15], which is located in the middle of the four α-helix bundle of each subunit. A third iron binding site has been found near this center in some archaeal and bacterial ferritins [5, 13, 16]. The FC has been found in all subunits of ferritins that are catalytically active except in the L subunit of mammalian ferritin, which is not catalytically active.

A third group of proteins in the ferritin-like superfamily which are able to store iron is the Dps family (miniferritins), which includes Dps and Dpr (Dps-like peroxide resistance) proteins. They have been found in bacteria [17, 18] and archaea [19]. Dps and Dpr proteins have 12 subunits that are assembled to form a spherical protein with tetrahedral symmetry (23 symmetry) and molecular mass of approximately 216 kDa. They can store up to about 500 iron atoms per protein molecule in their cavity [20]. It has been suggested that the physiological function of Dps is the protection of DNA from ROS rather than the storage of iron [21]. The diiron binding site of Dps is located at the interface between twofold-symmetry-related subunits. Like the FC in 24-meric ferritin, the FC of Dps is important for the functioning of the protein [22].

Because the structural genes for ferritin and Dps in *Pyrococcus furiosus* have no orthologs in the closely related species *Pyrococcus abyssi* and *Pyrococcus horikoshii*, we have searched for the presence of alternative iron-storage proteins in these organisms. Their genomes carry several genes encoding proteins that are members of the ferritin-like superfamily and which have ferritin-like and rubrerythrin-like domains. Homologs of these genes are present in *P. furiosus* as well [23]. Here we report on the product of one of these genes from *P. furiosus* (PF1196), which possibly shares a common ancestor with ferritins and rubrerythrins. The protein has iron-storage capacity comparable to that of ferritin and therefore we have named it archaeoferritin (AFR). Unlike ferritins, apo-AFR is purely monomeric. Addition of Fe(II) leads to oxidation by the protein and to oligomerization of monomers, which in turn affords the storage of the Fe(III) product in a mineral form comparable to the core in ferritin.

## Experimental procedures

### Chemicals

Anhydrous potassium hydrogen phosphate and potassium dihydrogen phosphate were purchased from J.T. Baker. All other chemicals (reagent grade) were purchased from Sigma-Aldrich.

### Protein expression and purification

2 ng of *P. furiosus* genomic DNA was used for PCR amplification of the PF1196 gene. To this goal, two primers were designed: CAGCCCATGGACATTGAAGAAG as a forward primer and ATTGGTACCTCACCGTGCTC as a reverse primer. The PCR product was purified and cloned in the pBAD/A (Invitrogen) vector using KpnI and NcoI restriction enzymes (New England Biolabs). The designed NcoI site in the forward primer brings about an Asn2 to Asp mutation, which by sequence comparison with ferritins was presumed to be nonconsequential. The clone obtained was sequenced (Baseclear, Leiden, The Netherlands), and after confirmation of the correct insert sequence the vector was used to transform TOP10 *Escherichia coli* (Invitrogen), the protein expression host strain. Cells were cultivated at 37 °C using terrific broth medium, and after 2 h (optical density at 600 nm of 0.6) production of the protein was induced with 0.02 % (final concentration) arabinose. After 8 h, cells were harvested using centrifugation and were broken using a cell disrupter (Constant Systems) operating at a pressure of 1.35 kbar. After centrifugation, the supernatant containing soluble proteins was collected. The final protein product was purified from *E. coli* proteins using a single heat step at 85 °C for 25 min. After centrifugation, the supernatant was extensively washed with 100 mM 3-(*N*-morpholino)propanesulfonic acid (MOPS) buffer pH 7.0, and was subsequently concentrated using Millipore filters, with a 10-kDa cutoff (Millipore). The final concentration of the protein was measured using the bicinchoninic acid assay reagent (Pierce) with bovine serum albumin as the standard.

### Preparation of apoprotein

Apoprotein was prepared by reduction of the as-isolated protein with sodium dithionite under a nitrogen (purity of 99.999 %) atmosphere using an Amicon stirred cell (Millipore). The working buffer was 100 mM MOPS pH 7 containing 100 mM NaCl. 2,2-Bipyridyl was used to chelate ferrous ions. The Fe(II)–dipyridyl complex was removed by consecutive dilution and concentration steps using an ultrafiltration membrane with a 10-kDa cutoff (Millipore). 100 mM MOPS pH 7.0 was used throughout this procedure. Apoprotein was found to contain less than 0.05 iron atoms per monomer.

### Iron determination

The ferene method [24] was used to determine the amount of residual iron in AFR. 100 μl of a protein solution of known concentration was mixed with 100 μL HCl (1 % w/v) and then the solution was incubated at 100 °C for 10 min. 100 μl Milli-Q water (Millipore) mixed with 100 μL HCl (1 % w/v) was used as the blank. Subsequently, 500 μl ammonium acetate (15 % w/v), 100 μl ascorbic acid (4 % w/v), and 100 μl sodium dodecyl sulfate (2.5 % w/v) were added to each sample. Finally, 100 μl (1.5 % w/v) of a solution of ferene (5,5′(3-(2-pyridyl)-1,2,4-triazine-5,6-diyl)-bis(2-furansulfonate)) was added to chelate the iron. The samples were centrifuged at 9,000 rpm for 5 min and the absorbance was measured at 593 nm.

### Iron oxidation

UV–vis spectra and progress curves of Fe(II) oxidation were measured using either an HP8453 spectrophotometer (Agilent) using 1-ml quartz cuvettes with a path length of 1 cm or a fiber-optic spectrophotometer (Avantes) using 1-ml glass cuvettes with a path length of 1 cm. Formation of Fe(III) was monitored at 315 nm after addition of an anaerobic solution of ferrous sulfate to the aerobic protein solution. The anaerobic ferrous sulfate solution was prepared as described previously [25]. Recording of spectra started less than 5 s after addition of ferrous sulfate. The iron oxidation reaction was conducted in 100 mM MOPS pH 7 containing 100 mM NaCl either at room temperature or at 47 °C. The Fe(II) oxidation kinetics experiments were performed at 47 °C to achieve complete oxidation of all added iron on a shorter timescale, and they were repeated with three different batches of protein. Each experiment was performed at least in duplicate.

### Oxygen consumption measurements

Oxygen consumption was measured amperometrically as described previously [25]. For each experiment a 4-μL aliquot of an anaerobic ferrous sulfate solution was added using a gastight syringe to a Clark-electrode cell (YSI) with a volume of 2 ml. The final protein concentration was 22 μM (monomer), and measurements were performed at 48 °C in 100 mM MOPS pH 7.0 containing 100 mM NaCl.

### Size-exclusion chromatography

Protein samples were applied to a Superdex 200 10/300 GL column (GE Healthcare) that was equilibrated with 100 mM MOPS pH 7.0 containing 0.15 M NaCl. For each measurement, a 200-μl sample was injected onto the column and the flow rate was 0.5 ml/min. Spectra were recorded with an HP 1040A spectrophotometer (Agilent). The column was calibrated using 480-kDa horse spleen ferritin (5 mg/ml), 67-kDa bovine serum albumin (8 mg/ml), 13.7-kDa bovine ribonuclease A (5 mg/ml), and 13.6-kDa horse cytochrome c (1.5 mg/ml).

### EPR spectroscopy

Spectra were recorded with a Bruker ECS-106 EPR spectrometer with the “Swedish” dewar system for sample cooling [26].

#### Circular dichroism (CD) and dynamic light scattering (DLS)

CD spectra were recorded using a JASCO-815 CD spectrometer in 0.2-ml quartz cuvettes with 1-mm path length. To minimize interference of the MOPS buffer, the buffer was changed to 100 mM phosphate buffer, pH 7.0. Dynamic light scattering (DLS) measurements were performed with a Zetasizer Nano ZS instrument (Malvern Instruments, UK) using 0.5-ml plastic cuvettes with 1-cm path length.

### Transmission electron microscopy

Transmission electron microscopy was performed with an FEI Tecnai TF20 electron microscope with a field emission gun as the source of electrons operated at 200 kV. The TEM sample was prepared by the addition of 50 Fe(II) ions per monomer to the AFR in 100 mM MOPS pH 7.0 containing 0.1 M NaCl. After 3 h incubation at room temperature, the sample was dialyzed against Milli-Q water overnight. A droplet of the sample was transferred to a Quantifoil^®^ carbon–polymer-coated copper grid. The sample was air-dried at room temperature prior to the measurements.

### High-mass matrix-assisted laser desorption/ionization time-of-flight mass spectrometry

Mass spectrometry measurements were performed using a matrix-assisted laser desorption/ionization (MALDI) time-of-flight (TOF) mass spectrometer equipped with CovalX’s HM2 high-mass detector system (CovalX, Zurich, Switzerland). For the control experiments, i.e., in the absence of cross-linking agent, 1 μl of protein (12 different concentrations of protein were used) was mixed with 1 μl of a sinapinic acid matrix (10 mg/ml) in acetonitrile/water (1:1, v/v) and 0.1 % trifluoroacetic acid (TFA). After mixing, 1 μl of each sample was spotted on the MALDI plate. The analysis was done in triplicate. After crystallization at room temperature, the plate was introduced into the MALDI mass spectrometer. For the cross-linking experiments, each mixture prepared for the control experiment [protein containing sinapinic acid matrix (10 mg/ml), acetonitrile/water (1:1, v/v), and TFA (1 %)] was submitted to cross-linking using CovalX’s K200 MALDI mass spectrometry analysis kit. From each sample, 9 μl was mixed with 1 μl of K200 stabilizer reagent (2 mg/ml). After incubation at room temperature (from 1 to 6 h), the samples were prepared for MALDI analysis as for control samples. Analysis was performed using the standard nitrogen laser and focusing on different mass ranges from 0 to 1,200 kDa. The parameter settings for the mass spectrometer were as follows: ion source 1, 20 kV; ion source 2, 17 kV (linear and positive mode); lens, 12 kV; pulse ion extraction, 400 ns. The HM1 high-mass detector parameters were as follows: gain, 2.25 kV; collision energy, 20 kV. The instrument was calibrated with clusters of insulin, bovine serum albumin, and IgG. For each sample three spots were analyzed (200 laser shots per spot). The results presented correspond to the sum of 200 laser shots. The mass spectrometry data were analyzed using Complex Tracker 2.0.

## Results

### Primary amino acid sequence analysis

**Fig. 1:**
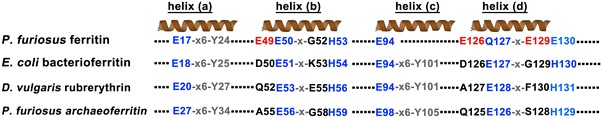
The putative ligands of the metal binding site in the ferritin-like superfamily of proteins. The ligands (or putative ligands) of a diiron binding site in *Pyrococcus furiosus* ferritin (PfFtn), *Escherichia coli* bacterioferritin, *Desulfovibrio vulgaris* rubrerythrin, and *P. furiosus* archaeoferritin (AFR) are shown in *blue*. The residues of a third iron binding site in *P. furiosus* ferritin are shown in *red*

The *P. furiosus* genome contains several genes encoding proteins that have been annotated as members of the ferritin-like superfamily of proteins, e.g., gene locus PF1196; these proteins have thus far remained uncharacterized. Analysis of the amino acid sequence of the protein product of PF1196, which we have named archaeoferritin (AFR), shows that it has 15 % identity (between residues 2 and 147) with that of rubrerythrin (*Desulfovibrio vulgaris*), 17 % identity (between residues 45 and 105) with that of bacterioferritin (*Rhodobacter capsulatus*), and 25 % identity (between residues 95 and 152) with that of manganese catalase (type II) (*Lactobacillus plantarum*). These results indicate that the secondary structure of protein is possibly composed of four α-helices; the helix A–B part exhibits 24 % identity with helix C–D. This internal evolutionary relationship has been observed for all members of the ferritin-like superfamily [3]. Alignment of the amino acid sequence of some members of the ferritin-like superfamily, i.e., ferritin, bacterioferritin, and rubrerythrin, with that of AFR suggests the presence of conserved motifs for a putative dimetal binding site in AFR similar to those of bacterioferritin and rubrerythrin: E-x_6_-Y (helix A), ExxH (helix B), E-x_6_-Y (helix C), and ExxH (helix D); see Fig. 1. The putative ligands of a third Fe(II) binding site, which has been observed in the X-ray crystal structure of *P. furiosus* ferritin (PfFtn) [5], are absent in AFR. The C-terminal domain of the protein lacks the rubredoxin-type mononuclear iron binding site (a single iron coordinated by four cysteines) found in rubrerythrin. The overall predicted secondary structure of AFR appears to be very similar to that of the crystallographically characterized members of the ferritin-like superfamily [5, 27]. In regular ferritins, 24 subunits assemble to form the quaternary structure of the protein: a hollow sphere with an internal cavity 8 nm in diameter. The molecular mechanism by which the monomers assemble to form the 24-meric nanocage of ferritin is not known. Because the amino acid sequence of AFR has less than 20 % identity with the amino acid sequences of ferritin and bacterioferritin, prediction of the quaternary structure of this protein by comparison of its amino acid sequence with that of ferritin is not possible. However, the X-ray crystal structure of a protein that shows more than 30 % identity with AFR, namely, the product of *P. furiosus* gene PF1190, has been solved (Protein Data Bank ID 2FZF) and shows that this protein is dimeric. Therefore, it may well be that AFR is also not 24-meric.

**Fig. 2:**
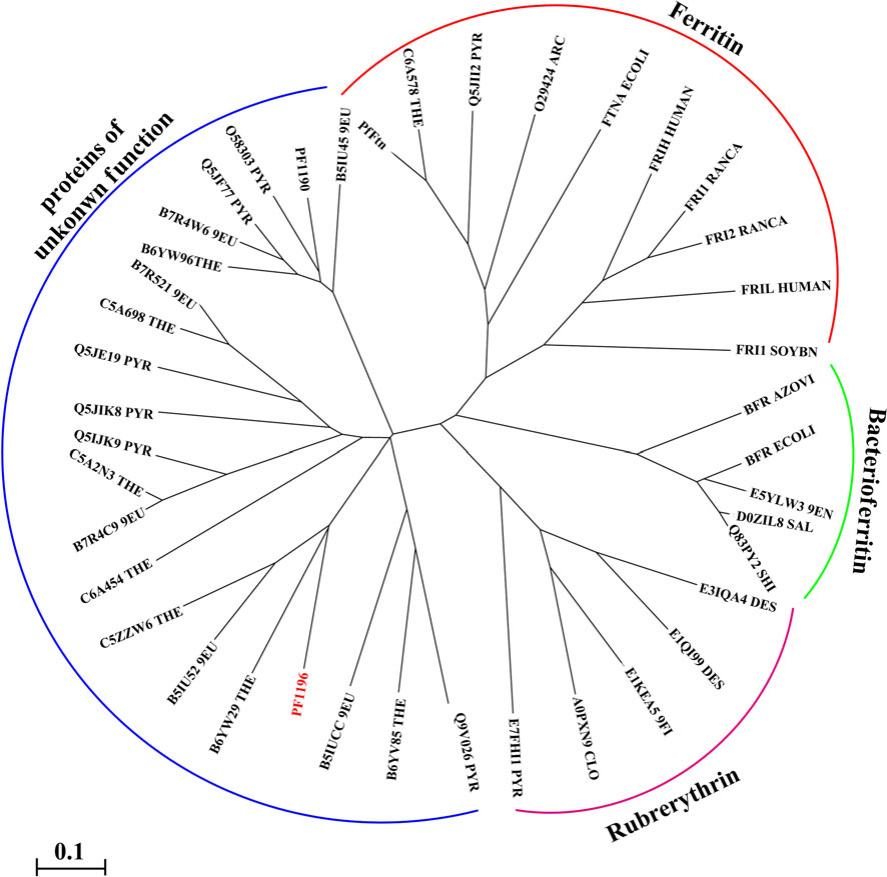
Unrooted phylogenetic tree showing the distance between AFR (gene locus PF1190) and its homologs in relation to ferritins and rubrerythrins. The sequence of *P. furiosus* AFR was compared with all sequences in the data bank using a BLAST search. Sequences with more that 30 % identity were used for multiple alignment with the sequence of ferritins, bacterioferritins, and rubrerythrins. The multiple sequence alignment and the phylogenetic tree were produced using the Phylip resource (http://bioweb.pasteur.fr)

For an overview of how AFR is distributed among species and how distant the sequences of AFR and its homologs are from those of ferritins and rubrerythrins, the amino acid sequence of *P. furiosus* AFR was compared with all the sequences in the data bank using a BLAST search. The sequences with an identity of 30 % or higher (*E* ≤ 10^−10^) were selected for multiple alignment with the amino acid sequences of ferritins and bacterioferritins, and the final outcome is shown as an unrooted phylogenetic tree in Fig. 2. The result indicates that AFR and its homologs represent a new subclass of the ferritin-like superfamily that appears to share a common ancestor with ferritins and rubrerythrins.

### Protein expression and characterization

**Fig. 3:**
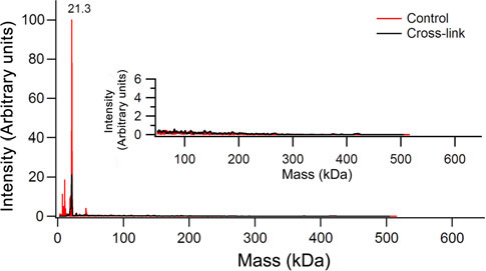
High-mass matrix-assisted laser desorption/ionization time-of-flight (MALDI-TOF) mass spectrometry of apo-AFR. The protein concentration was 1.8 μM monomer. The results are the average of three independent measurements, and each measurement comprises 200 combined laser shots. Samples were analyzed without cross-linking (control) (*red trace*) and with cross-linking (*black trace*)

The *P. furiosus* gene encoding PF1196 was cloned into the expression vector pBAD/A. Expression of the AFR in *E. coli* was achieved by induction with 0.02 % arabinose for 8 h at 37 °C. After a single-step purification by heating at 85 °C for 25 min and removal of the precipitated proteins by centrifugation, the protein was pure as determined by sodium dodecyl sulfate polyacrylamide gel electrophoresis, which showed a single band at the molecular mass of a monomer of approximately 21 kDa (Supplementary Fig. 1). In the as-isolated form, AFR contained about one iron per protein monomer, an amount comparable to that found in recombinantly produced ferritins [28]. Apo-AFR was produced as explained in “Experimental Procedures,” and its oligomeric state was determined by size-exclusion chromatography. In contrast to 24-meric ferritin, AFR was found to be mainly either monomeric or dimeric (Supplementary Fig. 2). To discriminate between these two possibilities, MALDI-TOF mass spectrometry was employed (Fig. 3). With apo-AFR in the presence or in the absence of cross-linking agent, only a single peak at a molecular mass of 21 kDa was observed, i.e., a monomer of AFR. Because in the presence of cross-linking agent the quaternary structure of proteins is retained during mass spectrometry measurement under the conditions used [29], it is concluded that the apo-AFR is predominantly monomeric. The hyperthermophilic archaeon *Archaeoglobus fulgidus* has been reported to produce an unusual ferritin that is mainly dimeric at low salt concentration and that upon the salt concentration being increased assembles into a 24-mer [30] with an unusual open pore structure [13]. Therefore, it was tested whether the oligomeric state of AFR is affected by NaCl concentrations from 50 to 500 mM. Unlike *A. fulgidus* ferritin, the oligomeric state of AFR was not affected by the salt concentration (not shown). The CD spectrum of as-isolated AFR was measured between 195 and 400 nm to determine the secondary structure of the protein (Supplementary Fig. 3). The spectrum is characteristic of a protein with a predominantly α-helical secondary structure as indicated by two negative peaks at 222 and 209 nm. The spectrum is similar to that of ferritin [31], and the results are consistent with the secondary structure prediction based on the amino acid sequence.

### Ferroxidase activity

**Fig. 4:**
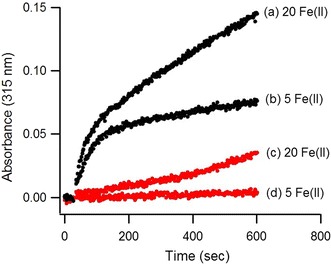
Fe(II) oxidation by AFR. An amount of ferrous iron corresponding to a stoichiometry of 5 and 20 Fe(II) ions per monomer was added to AFR. For the control experiments the same amount of iron was added in the absence of AFR. Traces *a* and *b* relate to the presence of 28.2 μM protein. Traces *c* and *d* relate to the absence of protein and show background oxidation of Fe(II) by molecular oxygen. The buffer was 100 mM 3-(*N*-morpholino)propanesulfonic acid (MOPS) pH 7.0 containing 100 mM NaCl. Measurements were made at room temperature

The iron oxidation activity, or ferroxidase activity, of AFR was measured by recording the progress curve of Fe(II) oxidation at 315 nm after addition of 5 or 20 Fe(II) ions per monomer in a single step to apoprotein at pH 7.0 and 22 °C (Fig. 4). Two phases were observed: a fast phase and a slow phase. The initial rate of each phase was faster than the background oxidation of Fe(II) by molecular oxygen (i.e., in the absence of protein), confirming that the measured activity was due to oxidation of Fe(II) catalyzed by AFR. At the end of the catalyzed reaction, no Fe(III) precipitate was formed, which may suggest that the Fe(III) product was stored by AFR in a soluble form comparable to core formation in ferritins.

The ferroxidase activity of AFR was also assessed using a different method by measuring oxygen consumption. An amount of 4 Fe(II) ions per monomer was added to the apoprotein at pH 7, and oxygen consumption was measured at 48 °C. A stoichiometry of 0.28 ± 0.02 molecules of dioxygen consumed per Fe(II) ion oxidized was found. A similar stoichiometry has been observed for bacterioferritin [32]. A stoichiometry of 0.25 molecular oxygen per Fe(II) ion is consistent with the production of water.

### Evidence for the presence of a diiron binding site

**Fig. 5:**
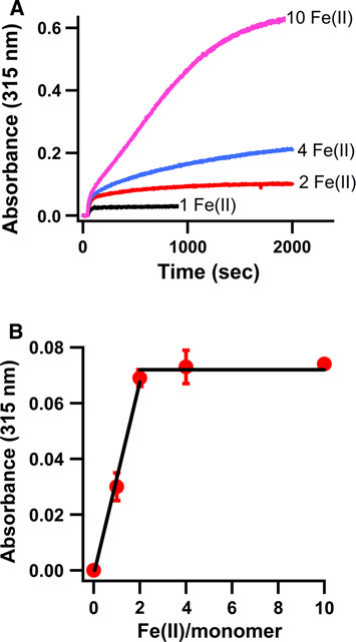
UV–vis spectroscopic evidence for the presence of a diiron binding site. **a** Progress curve of Fe(III) formation by AFR after addition of 1, 2, 4, and 10 Fe(II) ions per monomer to apoprotein and **b** the maximum absorbance at the end of the fast phase for these progress curves versus the number of Fe(II) ions per monomer of AFR. Progress curves were measured at 315 nm in 100 mM MOPS pH 7.0 containing 100 mM NaCl. The protein concentration was 25 μM. All measurements were made at 45 °C

Because sequence comparison of AFR with the ferritin-like proteins suggests the presence of a diiron binding center in AFR, and because the progress curves of Fe(II) oxidation showed a fast phase and a slow phase, reminiscent of the kinetics of PfFtn [25] and *E. coli* bacterioferritin [32], it was determined whether the fast phase reflected oxidation of 2 Fe(II) ions per monomer as an indication of Fe(II) oxidation at an apoferroxidase center. Amounts of ferrous iron corresponding to stoichiometries of 1, 2, 4, or 10 Fe(II) ions per monomer were added to apo-AFR and progress curves were recorded at 315 nm. Measurements were made at 45 °C to accelerate the slow oxidation phase and achieve substrate depletion over a shorter timescale. Figure 5 shows that up to complete oxidation of 2 Fe(II) ions per monomer, the maximum absorbance of the fast phase increased linearly with the amount of added Fe(II) per monomer. However, addition of 4 or 10 Fe(II) ions per monomer did not change the maximum absorbance at the end of the fast phase. The slow oxidation phase was observed for Fe(II) per monomer ratios greater than 2 and was negligible for ratios less than 2.

The molar extinction coefficient of Fe(III)-bound AFR was measured for iron loading between 0 and 10 Fe(II) ions per monomer. The absorbance at 315 nm was plotted versus the amount of Fe(II) per monomer (Supplementary Fig. 4). Linear fits were obtained only when the data for 0–2 iron ions and 2–10 iron ions were analyzed separately. Therefore, it appears that the first two Fe(III) ions per monomer in AFR, which we presume to reside in the ferroxidase center, have a molar extinction coefficient (1.6 mM^−1^ cm^−1^) different from that of the mineralized iron in the putative core of AFR (2.4 mM^−1^ cm^−1^). This is comparable to the observation that the two Fe(III) ions in the FC of *E. coli* bacterioferritin have a molar extinction coefficient different from that of Fe(III) in the core [32].

### Formation of a ferritin-like mineral core

**Fig. 6:**
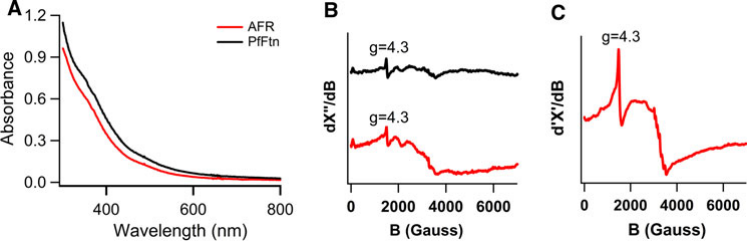
Spectroscopy of Fe(III)-loaded protein. **a** Optical spectrum of AFR containing 20 Fe(III) ions per monomer in comparison with that of PfFtn containing 500 Fe(II) ions per 24-mer. The AFR concentration was 39.5 μM (monomer) and the PfFtn concentration was 36.5 μM (monomer). **b** EPR spectra of 640 μM (monomer) AFR: from top to bottom apo-AFR and AFR containing 40 Fe(III) ions per monomer. **c** EPR spectrum of 540 μM (monomer) PfFtn containing 50 Fe(III) ions per monomer. The EPR conditions were as follows: microwave frequency, 9.24 GHz; microwave power, 200 mW; modulation frequency, 100 kHz; modulation amplitude, 12.5 G; temperature, 110 K

To verify that the AFR has the ability to store Fe(III) in a manner similar to that of ferritin, apo-AFR was aerobically incubated with an amount of ferrous iron corresponding to a stoichiometry of 20 Fe(II) ions per monomer for 3 h at room temperature and subsequently the UV–vis spectrum of the sample was measured between 300 and 800 nm and compared with the spectrum of PfFtn aerobically loaded with the same amount of Fe(II) per monomer, as shown in Fig. 6a. The spectrum of the loaded AFR is very similar to that of Fe(III)-loaded PfFtn, and thus suggests that a ferrihydrite-like mineral core was formed. The molar extinction coefficient of 2.4 ± 0.2 mM^−1^ cm^−1^ at 315 nm for the putative AFR core is close to the reported values for the core of PfFtn (2.5 mM^−1^ cm^−1^ at 315 nm) [25] and human H-chain ferritin (2.1 mM^−1^ cm^−1^ at 305 nm) [33].

The efficiency of Fe(III) storage by AFR was compared with that by PfFtn as follows. 30 Fe(II) ions per monomer in six steps, in each step 5 Fe(II) ions per monomer, were added to AFR or PfFtn (24-mer). After incubation at room temperature for at least 3 h, samples were concentrated by ultrafiltration using a membrane with a cutoff of 10 kDa. Subsequently, the amount of Fe(III) in the filtrate was measured using the ferene method. It was found that both PfFtn and AFR were able to store more than 99 % of the added iron. Also, no formation of insoluble Fe(III) precipitate was observed, even with addition of larger amounts of Fe(II) up to a stoichiometry of 50 Fe(II) ions per monomer.

Electron paramagnetic resonance (EPR) spectroscopy was used to explore the chemical form of the iron stored in the protein. 50 Fe(II) ions per monomer in five steps, in each step 10 Fe(II) ions per monomer, were aerobically added to AFR and as a reference to PfFtn, and the samples were incubated at room temperature for at least 5 h with stirring to allow complete oxidation of Fe(II) by the protein. Subsequently, the EPR spectra of the samples were recorded. The *g* = 4.3 EPR signal of mononuclear Fe(III) was negligible in the case of AFR. A broad signal (Fig. 6b) reminiscent of the PfFtn core signal (Fig. 6c) was observed at 110 K. The intensity of the signal of the AFR core was significantly less than that of the PfFtn core, but in comparison the signal was broader. The spectrum of the AFR core shows detailed features of unknown origin. The signal disappeared at temperatures below 50 K. This indicates that at temperatures below 50 K the crystalline domains of the ferrihydrite-like mineral core of AFR or PfFtn become magnetically ordered, suggesting that the nanoparticle stored in AFR is superparamagnetic just like the core in PfFtn [28].

Formation of a ferritin-like mineral core was further confirmed by transmission electron microscopy. TEM was applied to AFR and as a reference to PfFtn containing 50 Fe(III) ions per monomer (Supplementary Fig. 5). TEM of the AFR sample showed a distribution of different core sizes. The maximum core size was 5–7 nm, which is slightly smaller than the size of the PfFtn core (8 nm). No aggregation and formation of larger particles was observed in both samples.

Circular dichroism spectroscopy was used to monitor changes in the secondary structure of AFR upon binding and oxidation of Fe(II). AFR containing 30 Fe(III) ions per monomer in 100 mM MOPS pH 7.0 was buffer-exchanged to 100 mM phosphate buffer pH 7.0 to eliminate the contribution of MOPS to the spectrum. The CD spectrum was measured between 400 and 190 nm. No significant differences in the secondary structure as compared with the apoprotein were found (Supplementary Fig. 6).

### Reversible assembly of monomers upon binding and oxidation of Fe(II)

**Fig. 7:**
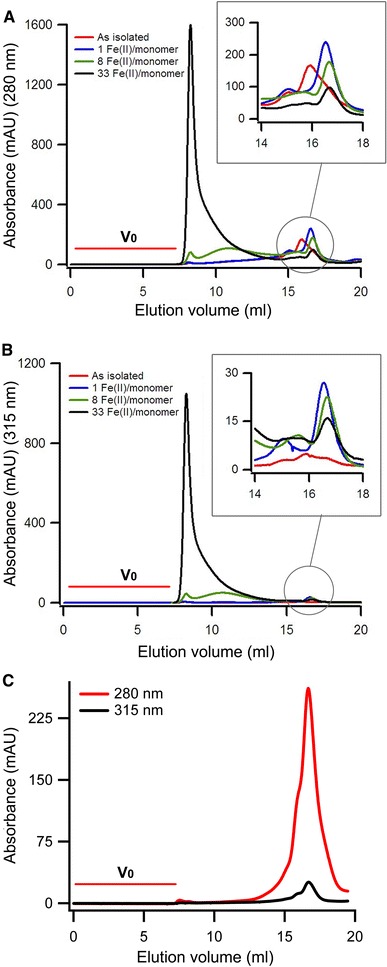
Size-exclusion chromatography of Fe(III)-loaded AFR. As-isolated protein was incubated aerobically with different amounts of Fe(II), and after complete oxidation of iron, size-exclusion chromatography was used to measure the formation of multimeric structures. **a** Chromatogram at 280 nm and **b** chromatogram at 315 nm. **c** Chromatogram at 280 and 315 nm of apo-AFR produced after chemical reduction and chelation of iron from AFR that contained 30 Fe(III) ions per monomer. The AFR concentration was 48 μM (monomer). The buffer was 100 mM MOPS pH 7.0 containing 0.1 M NaCl. The flow rate was 0.5 ml/min. *V*_0_ indicates the void volume of the column

A possible operational mechanism for iron oxidation and storage by AFR involves assembly of monomers to form nanocages with a structure similar to that of the ferritin nanocage. To investigate this, monomers were loaded aerobically with different amounts of Fe(II) and size-exclusion chromatography was used to monitor changes in the molecular size of the protein. Chromatograms were recorded at 280 and 315 nm to monitor protein and Fe(III), respectively. Figure 7a and b shows that AFR containing 1 Fe(III) ion per monomer was exclusively monomeric. Addition of one Fe(II) ion per monomer changes the hydrodynamic properties of the protein as the peak of the monomer shifts slightly to a lower molecular weight. Protein containing more than 2 Fe(III) ions per monomer consisted of oligomers with different sizes, and the amount of monomeric protein decreased concomitantly as the amount of Fe(II) added increased. The average size of the oligomers increased as the iron content of the protein increased. Reversibility of the association of monomers and formation of multimers with a ferric mineral core was determined as follows. Multimeric AFR that contained 30 Fe(III) ions per monomer was converted to apo-AFR by chemical reduction and chelation of Fe(II) as described in “Experimental Procedures.” Size-exclusion chromatography of the apoprotein produced in this manner exhibited a single peak (as observed with the as-isolated protein), implying that protein had returned to its original monomeric form as apoprotein (Fig. 7c), and did not exhibit the small fraction of oligomeric structures which had been observed after preparing apoprotein from the as-isolated AFR (Supplementary Fig. 2). The small fraction of oligomeric structures that is observed in Supplementary Fig. 2 is presumably due to the presence of a few Fe(III) ions. The regenerated monomeric protein was able to oxidize Fe(II) again and to assemble into multimers with storage of Fe(III).

The uniformity and size distribution of the multimeric structures formed after binding and oxidation of Fe(II) were measured using dynamic light scattering (DLS). As-isolated protein was loaded with 0, 10, 30, and 50 Fe(III) ions per monomer at 55 °C and the size distribution of the protein nanocages including their mineral core was measured with DLS at 25 °C (Supplementary Fig. 7). The results confirm that the average size of these nanocages increases with increasing amounts of Fe(III). For the AFR sample containing 50 Fe(III) ions per monomer, the hydrodynamic diameter of more than 90 % of the particles was between 18 and 38 nm. Dynamic light scattering of the reference sample—PfFtn containing 50 Fe(III) ions per monomer—shows that the diameter of more than 90 % of the particles was between 8 and 18 nm (Supplementary Fig. 7). Thus, loading of the AFR resulted in formation of protein nanocages with a broader size distribution compared with the size distribution of the ferritin nanocage. This is perhaps because the ferritin nanocage is preassembled and loading of the ferritin with Fe(II) leads to formation of particles with uniform sizes (8 nm) inside the cavity of ferritin, whereas the monomers of the AFR assemble upon binding and oxidation of Fe(II), which generates oligomers with different numbers of subunits and thus particles with different hydrodynamic diameters.

**Fig. 8:**
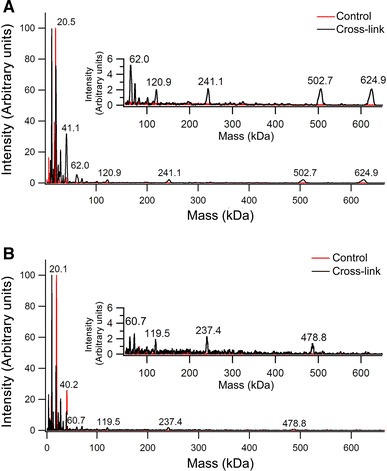
Monomers of AFR form oligomeric structures upon oxidation of Fe(II). High mass MALDI-TOF mass spectrometry of **a** 1.7 μM (monomer) AFR containing 50 Fe(III) ions per monomer, and **b** 7.3 μM (24-mer) apo-PfFtn. The results are the average of three independent measurements, and each measurement comprises 200 combined laser shots. Samples were analyzed without cross-linking (control) (*red trace*) and with cross-linking (*black trace*)

To gain more detailed insight into the assembly of the monomers of AFR upon binding and oxidation of Fe(II), MALDI-TOF mass spectrometry was performed on AFR containing 50 Fe(III) ions per monomer. The results are compared with those of an apo-PfFtn reference sample in Fig. 8. As observed before in chromatography (see earlier), the high-mass MALDI-TOF mass spectrometry measurements of apo-AFR in the presence and absence of cross-linking showed a single peak at 21 kDa corresponding to monomeric protein (Fig. 3). Subsequently, the measurements were performed with AFR containing 50 Fe(III) ions per monomer and with apo-PfFtn as a reference. Different oligomeric structures with defined masses were observed in the samples that were treated with the cross-linking agent. For PfFtn these masses were 480 kDa (24-mer), 240 kDa (12-mer), 120 kDa (hexamer), and 40 kDa (dimer) and for AFR containing 50 Fe(III) ions per monomer the masses were 624 and 500 kDa (24-mer), 240 kDa (12-mer), 120 kDa (hexamer), 62 kDa (trimer), and 41 kDa (dimer). The 624-kDa peak can be assigned to an oligomeric structure with more than 24 subunits, or to a 24-meric structure plus the iron core. When the cross-linking reaction was omitted, only a peak at 21 kDa was observed for both AFR and PfFtn. Because, PfFtn is a 24-mer (480 kDa) in its native form, the results show that the presence of a cross-linking reagent is essential for the observation of the quaternary structure of the protein with high-mass MALDI-TOF mass spectrometry [29]. Furthermore, it is clear that AFR forms discrete oligomeric structures comparable to those of PfFtn.

## Discussion

We have heterologously expressed, purified, and characterized the protein product of a gene in *P. furiosus* (PF1196) which has similarity to the iron-storage proteins of the ferritin-like superfamily. Phylogenetic analysis shows that it is a member of the ferritin-like superfamily of proteins and indicates that it possibly shares a common ancestor with ferritin, bacterioferritin, and rubrerythrin. Biochemical studies show that its structure consists of a four α-helix bundle domain comparable to those of ferritin and rubrerythrin. Although ferritins are preassembled 24-meric proteins that store iron in their internal cavity, in vitro characterization of the novel protein shows that in the absence of iron the protein is monomeric. Upon oxidation of iron, oligomeric structures are formed that store the iron in a mineral core which has spectroscopic properties comparable to those of ferritin. Therefore, the protein is named *P. furiosus* archeaoferritin (AFR). *P. furiosus* AFR has close orthologs (more than 30 % identity) in *P. abyssi* and *P. horikoshii*.

The iron storage and concomitant association of monomers into multimeric AFR is a reversible process. The reversible nature of the assembly of monomeric AFR is different from previously reported salt-induced irreversible association of dimeric forms of *Deinococcus radiodurans* Dps [34] or the salt-dependent association of dimeric forms of *A. fulgidus* ferritin [30]. Detailed structural information is required to understand why AFR is unable to form a multimeric structure similar to ferritins in the absence of iron.

The observation of iron-induced reversible association of the monomers of AFR suggests a novel mechanism of iron storage and formation of oligomeric structures among the ferritin-like superfamily of proteins. We propose that this mechanism is possibly an alternative way to prevent toxicity of free Fe(II) and molecular oxygen and simultaneously to store Fe(III) in a soluble and biologically available form in organisms that lack the 24-meric ferritin and/or the 12-meric Dps, such as the close relatives of *P. furiosus*: *P. abyssi*, and *P. horikoshii*. It is not known why *P. furiosus* has an apparent redundancy in genes encoding iron-storage proteins as it encodes AFR (gene locus PF1196), ferritin (gene locus PF0742), and Dps (gene locus PF1193). The structural gene of AFR in *P. furiosus* is located in what appears to be a cluster of genes whose translated sequences suggest involvement in iron homeostasis, including the genes encoding Dps and Fur (ferric uptake regulator, gene locus PF1195).

## Electronic supplementary material

Below is the link to the electronic supplementary material.

Supplementary material 1 (PDF 323 kb)

## Abbreviations

AFR: Archaeoferritin
CD: Circular dichroism
DLS: Dynamic light scattering
EPR: Electron paramagnetic resonance
MALDI: Matrix-assisted laser desorption/ionization
MOPS: 3-(*N*-Morpholino)propanesulfonic acid
PfFtn: *Pyrococcus furiosus* ferritin protein
ROS: Reactive oxygen species
TEM: Transmission electron microscopy
TOF: Time-of-flight
