# Supplementary material for: A novel mechanism of iron-core formation by Pyrococcus furiosus archaeoferritin, a member of an uncharacterized branch of the ferritin-like superfamily
Source: J Biol Inorg Chem. 2012 Jun 28;17(6):975–85. doi: 10.1007/s00775-012-0913-0 (PMC3401498; doi:10.1007/s00775-012-0913-0)
Supplement: Supplementary file 1 — Supplementary material 1 (PDF 323 kb) [file 775_2012_913_MOESM1_ESM.pdf]

SUPPLEMENTAL DATA

A novel mechanism of iron-core formation by *Pyrococcus furiosus* archaeoferritin, a member of an uncharacterized branch of the ferritin-like superfamily

Kourosh Honarmand Ebrahimi, Peter-Leon Hagedoorn, Laura van der Weel, Peter D.E.M. Verhaert, Wilfred R. Hagen

Department of Biotechnology, Delft University of Technology, Julianalaan 67, 2628BC Delft, The Netherlands

Address correspondence to: Wilfred R. Hagen, Department of Biotechnology, Delft University of Technology, Julianalaan 67, 2628BC Delft, The Netherlands. Phone: +31152785051 ; Fax: +31152782355; Email: w.r.hagen@tudelft.nl

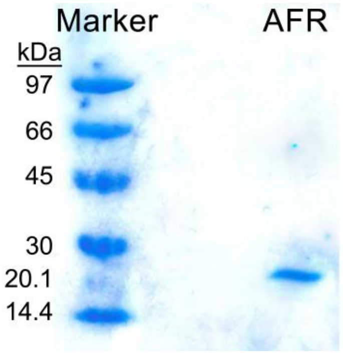

Figure S1. SDS gradient gel electrophoresis of as-isolated *Pyrococcus furiosus* archaeoferritin (AFR) after purification showing a single band at circa 21 kDa.

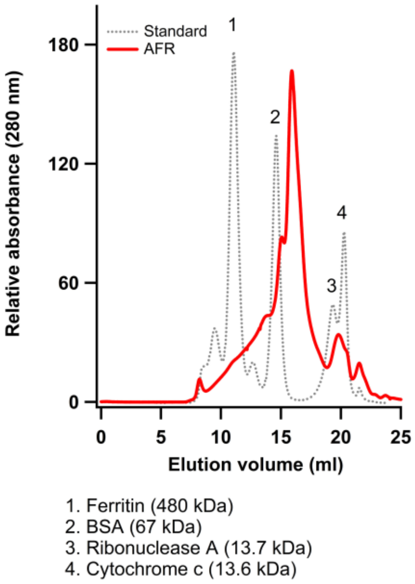

Figure S2. Determination of the size of *P. furiosus* archaeoferritin. Size exclusion chromatogram of 48 µM apo-protein (monomer) at pH 7.0, 0.1 M NaCl. Flow rate was 0.5 ml/min. The small fraction of oligomeric structures is presumably due to the presence of a few Fe(III).

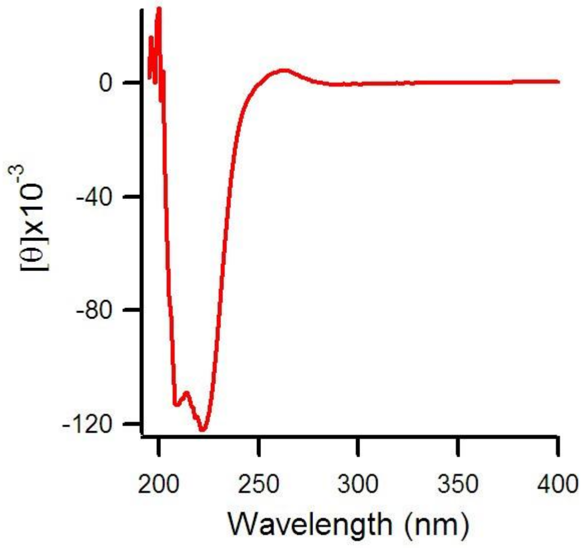

Figure S3. Circular dichroism spectrum of 45 µM (monomer) as-isolated *P. furiosus* archaeoferritin in 100 mM phosphate buffer, pH 7.2.

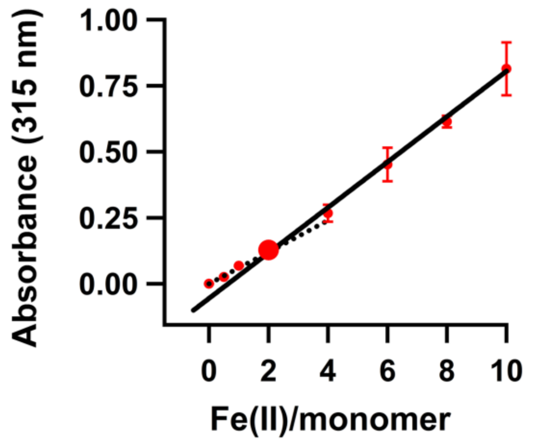

Figure S4. UV-visible spectroscopy of Fe(II) titration of *P. furiosus* AFR. Final absorbance at 315 nm is plotted versus amount of Fe(II) added aerobically to AFR protein. The broken line is a linear fit for 0-2 Fe(II) per monomer and the solid line shows the linear fit for 2-10 Fe(II) per monomer. Protein concentration was 40.8 µM (monomer). Buffer was 100 mM Mops, pH 7.0, 100 mM NaCl. Measurements were done at room temperature. Two independent measurements were used to calculate standard deviation of each point. The incubation time for all samples was about 1 hr at 40 ºC and 4 hr at room temperature.

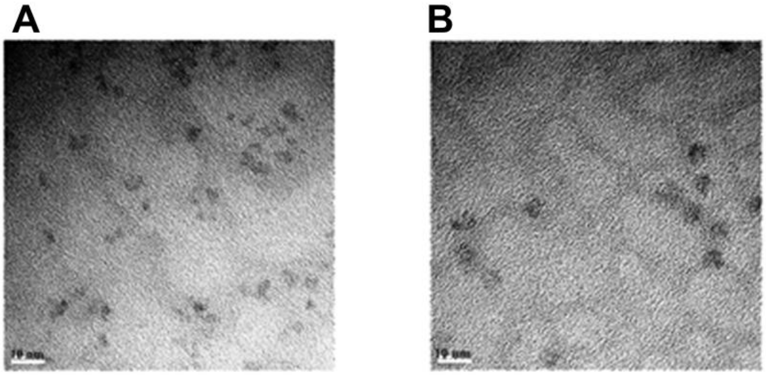

Figure S5. Formation of ferrihydrite like core in *P. furiosus* AFR. Transmission electron microscopy (TEM) recording of (A) 50 Fe(II) per monomer loaded AFR. Monomer concentration was 49 µM. (B) 50 Fe(II) per monomer aerobically loaded sample of *P. furiosus* ferritin. Concentration of protein was 2.8 µM. Iron was added in 100 mM Mops, pH 7.0, 0.1 M NaCl. After this, the solution was dialyzed against Milli-Q water. Scale-bar = 10 nm.

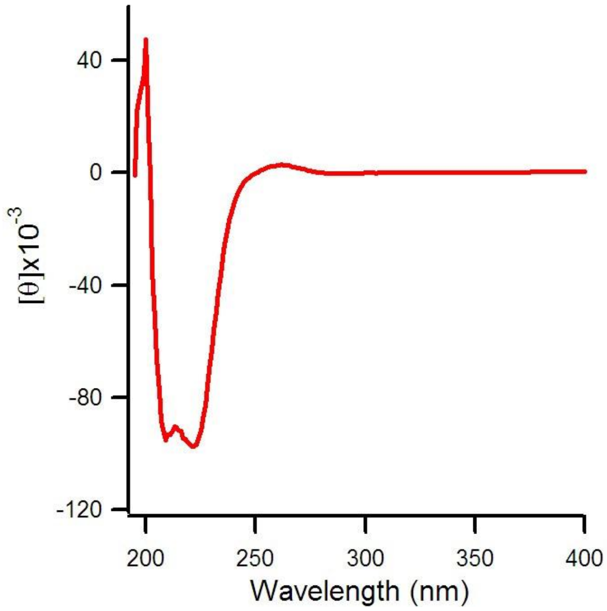

Figure S6. CD spectrum of as isolated *P. furiosus* AFR after aerobic addition of 25 Fe(II) per monomer. Concentration of protein was 40 µM (monomer). Protein was in 100 mM phosphate buffer, pH 7.2.

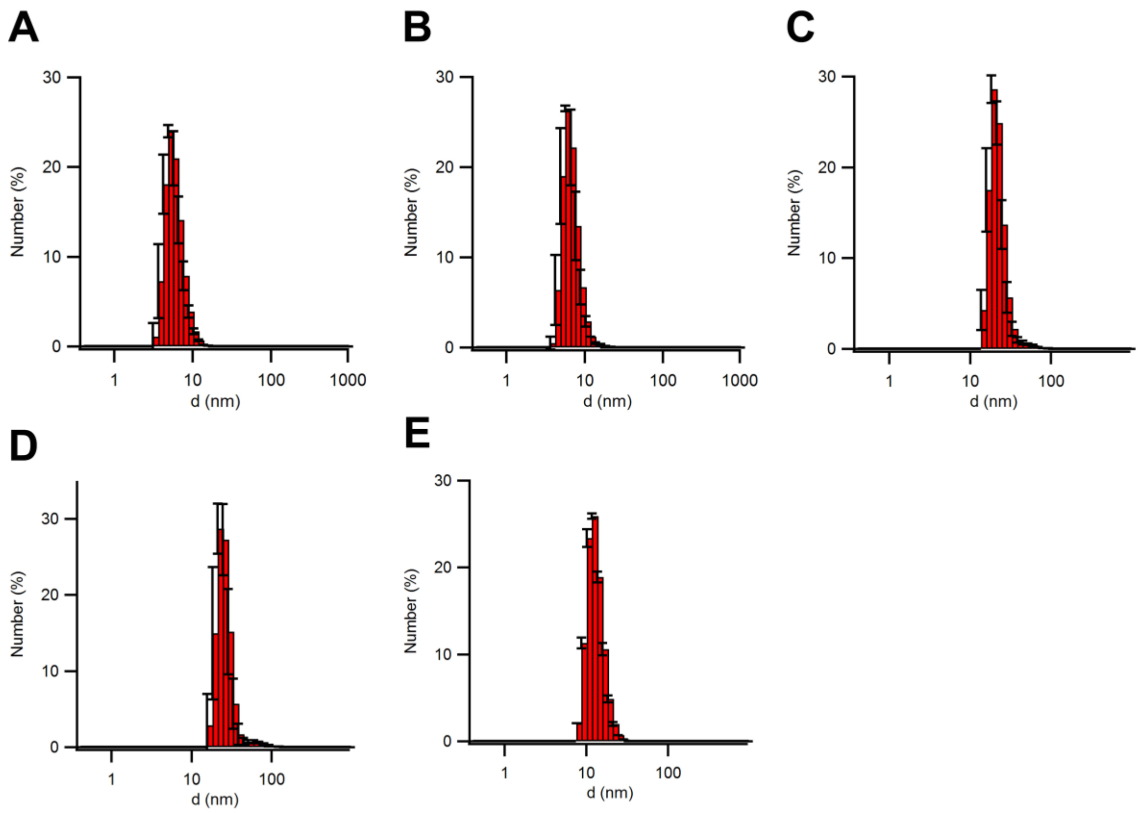

Figure S7. Dynamic light scattering of *P. furiosus* AFR and *P. furiosus* ferritin. Comparison of size distribution of AFR loaded with different amounts of Fe(II) and ferritin. Size distribution of (A) as isolated protein, (B) apo protein loaded with 10 Fe(II) per monomer, (C) apo protein loaded with 30 Fe(II) per monomer, and (D) apo protein loaded with 50 Fe(II) per monomer. Protein concentration (monomer) was 28.8 µM. (E) Size distribution of apo-ferritin loaded with 50 Fe(II) per monomer. Protein concentration of 24-mer ferritin was 3.8 µM. Buffer was 100 mM Mops, pH 7.0, and 0.1 M NaCl.
